# Tumor-Infiltrating Cytotoxic T Cells and Tumor-Associated Macrophages Correlate With the Outcomes of Neoadjuvant Chemoradiotherapy for Locally Advanced Rectal Cancer

**DOI:** 10.3389/fonc.2021.743540

**Published:** 2021-10-18

**Authors:** Yuqin Yang, Wenjing Tian, Liqian Su, Peiqiu Li, Xiaohua Gong, Lu Shi, Qingling Zhang, Bin Zhao, Hong Zhao

**Affiliations:** ^1^ The Cancer Center of The Fifth Affiliated Hospital of Sun Yat-Sen University, Zhuhai, China; ^2^ Department of Pathology, Guangdong Provincial People’s Hospital, Guangdong Academy of Medical Sciences, Guangzhou, China; ^3^ Department of Pathology, School of Basic Medical Science, Southern Medical University, Guangzhou, China; ^4^ Guangdong Provincial Key Laboratory of Biomedical Imaging, The Fifth Affiliated Hospital, Sun Yat-sen University, Zhuhai, China; ^5^ Precision Medicine Center of Harbin Medical University Cancer Hospital, Harbin, China; ^6^ Department of Nephrology, The Fifth Hospital Affifiliated of Sun Yat-sen University, Zhuhai, China; ^7^ The Second Affiliated Hospital and Yuying Children’s Hospital of Wenzhou Medical University, Wenzhou, China

**Keywords:** cytotoxic T lymphocytes, tumor-associated macrophages, rectal cancer, neoadjuvant chemoradiotherapy, pathological complete response

## Abstract

**Background:**

Tumor-infiltrating immune cells (TIICs) play a key role in immunoregulatory networks and are related to tumor development. Emerging evidence shows that these cells are associated with sensitivity to chemotherapy and radiotherapy. However, the predictive role of TIICs in the outcomes of neoadjuvant chemoradiotherapy (nCRT) for locally advanced rectal cancer (LARC) is unclear.

**Methods:**

Imaging mass cytometry (IMC) was performed to comprehensively assess the immune status before nCRT in 6 patients with LARC (3 achieved pathological complete response (pCR), 3 did not) with matched clinicopathological parameters. Immunohistochemistry (IHC) for CD8, CD163 and Foxp3 on biopsy samples from 70 patients prior to nCRT and logistic regression analysis were combined to further evaluate its predictive value for treatment responses in an independent validation group.

**Results:**

A trend of increased CD8+ cytotoxic T lymphocytes (CTLs) and decreased CD163+ tumor-associated macrophages (TAMs) and Foxp3+ regulatory T cells (Tregs) in the pCR group was revealed by IMC. In the validation group, CTLs and TAMs were strong predictors of the clinical response to nCRT. High levels of CTLs were positively associated with the pCR ratio (OR=1.042; 95% CI: 1.015~1.070, p=0.002), whereas TAMs were correlated with a poor response (OR=0.969; 95% CI: 0.941~0.998, p=0.036). A high density of TAMs was also associated with an advanced cN stage.

**Conclusion:**

CTLs in the tumor microenvironment (TME) may improve the response to nCRT, whereas TAMs have the opposite effect. These results suggest that these cells might be potential markers for the clinical outcomes of nCRT and aid in the clinical decision-making of LARC for improved clinical outcomes.

## Introduction

Rectal cancer treatment depends on various factors, including clinical stage, pathological grade, and patient age. For patients who are diagnosed with locally advanced rectal cancer (LARC), preoperative 5-fluorouracil-based chemoradiotherapy (CRT), followed by surgery, has been recommended as the standard treatment (1). Neoadjuvant CRT (nCRT) has been demonstrated to reduce tumor size, make it easier to remove, and reduce the risks of local recurrence and distant metastasis (2). Importantly, pathological complete response (pCR), defined as no remaining visible tumor cells in the surgical specimen on a histopathologic assessment, has already been proven to be associated with prolonged disease-free survival (DFS) and overall survival (OS) (3). Unfortunately, the pCR ratio is only approximately 10-20% in LARC patients who receive nCRT, and most of the patients experience only mild to moderate tumor regression or even progression following nCRT (4). Numerous studies have been carried out to explore the mechanisms of the clinical response to chemoradiotherapy (5–7), but the biomarkers that represent nCRT sensitivity remain poorly understood; thus, further studies are needed to improve the pCR ratio of nCRT.

Recently, emerging evidence has shown that the antitumour effect of chemotherapy and radiotherapy is related to serious immune-related mechanisms (8). CRT was proven to improve the suppressive tumor microenvironment (TME) by suppressing or eliminating immunosuppressive cells (8, 9). as well as prompt tumor cells to release neoantigens, which results in the activation and proliferation of cytotoxic T lymphocytes (CTLs) (10). Respectively, the association between tumor-infiltrating immune cells (TIICs) and sensitivity of nCRT got extensive attention (11, 12). In breast cancer, circulating MDSC and Treg subsets were higher in non-responders than responders in patients after nCRT (9). Although studies above have attempted to investigate the role that TIICs play in the process of nCRT, their predictive value in LARC still needs to be confirmed through more studies.

The tumor immune microenvironment (TIME) is a spatially organized but complex ecosystem that is comprised of multiple immune cell types. To interrogate the complexity of the TIME, technologies were created to gauge the expression of multiple proteins within a single cell. However, for routine laboratory assays, such as flow cytometry, the tissue must be dissociated into a cell suspension, thus leading to the loss of architecture, which contains important bioinformatics information. This dilemma drove the development of imaging mass cytometry (IMC), which can solve the problems mentioned above (13, 14). IMC makes use of heavy metal-conjugated antibodies that are ionized from the surface of a tissue slide. Since rare-earth metals are not found in biological tissues, IMC circumvents the spectral overlap limitation in flow cytometry and allows a simultaneous distinguishment of over 50 parameters at the single-cell level. In addition, IMC retains spatial information, making it possible to reveal tissue context and cellular interactions that show distinct architecture in physiological and disease states. Because of its superior advantages, IMC has been popularly applied for the immunophenotyping of TMEs. This has greatly promoted the discovery of rare cell subsets and the assessment of the relationship between cellular phenotype diversity and therapeutic outcomes. Currently, IMC has been extended to mechanistic studies beyond phenotypic observations (15).

In the present study, to comprehensively assess the immune status of LARC and its correlation to clinical outcomes after nCRT, we developed a 12-antibody panel containing the major TIIC subsets (including CD4+ T cells, CD8+ CTLs, Tregs, NK cells, B cells, monocytes, and tumor-associated macrophages (TAMs)) for the analysis of LARC biopsy samples by IMC. Immunohistochemistry (IHC) and logistic regression analysis were adopted for independent validation in a larger group. The current study aimed to determine whether the density of infiltrating immune cells pre-nCRT was correlated with the subsequent treatment response and to allow more rational therapeutic strategies to be developed in the future.

## Methods

### Patients

This study retrospectively enrolled 76 patients (6 in the training group, 70 in the validation group) with LARC who received nCRT at the Fifth affiliated Hospital of Sun Yat-sen University and Harbin Medical University Cancer Hospital between December 2011 and September 2017. The inclusion criteria were as follows: (1) complete information on medical records and pathological confirmation of LARC; (2) stage II/III disease by MRI or CT combined with endorectal ultrasound according to the eighth edition of the American Joint Committee on Cancer (AJCC) Staging Manual; (3) no previous history of cancer surgery, pelvic radiotherapy or systemic chemotherapy; and (4) no other history of a malignant tumor; (5) No patient had autoimmune diseases, such as rheumatoid diseases, or other serious diseases.

The immune statuses of six patients (3 who achieved pCR, 3 who did not) with matched clinicopathological parameters were determined by IMC. We further enrolled 70 patients [14 who achieved pCR, 56 who did not (all patients achieved partial response or stable disease, no progressive disease)] with LARC in this study for independent validation; a representative formalin-fixed, paraffin-embedded (FFPE) sample was selected from each rectal biopsy sample for immunohistochemical staining.

This experiment was approved by the Ethics Committee of the Fifth affiliated Hospital of Sun Yat-sen University and Harbin Medical University Cancer Hospital, and written informed consent was obtained from all patients.

### IMC

Four-micrometer-thick FFPE sections were stained with a panel of 12 antibodies (**Table 1**). Briefly, tissue sections were dewaxed with xylene and rehydrated sequentially with 100% to 70% ethanol before cleaning with PBS. Heat-induced antigen retrieval was performed in a pressure cooker for 9 min in Tris-EDTA buffer (pH 9.0). The slides were cooled to room temperature (RT) and then blocked at RT with 3% BSA for 1 h. At the same time, the antibody panel was prepared with the antibody diluent solution. Each slide was incubated overnight with all antibodies at 4°C. The next day, the slides were washed with PBS containing 0.2% Triton-X 3 times, and DNA was labelled with Intercalator-Ir (1:400 dilution) for 30 min at RT. Before IMC acquisition, the slides were rinsed with ddH_2_O for 10 min and air dried for at least 20 min. The IMC assay was purchased from Fluidigm. All IMC steps were performed in accordance with instructions from the manufacturer. An area of approximately 500 × 500 μm was selected based on bright field images. Two or three regions of interest (ROIs) were selected for each slide, depending on the size of the section. After the Hematoxylin-eosine(HE) staining, the areas with the highest concentration of immune cells were selected as ROIs. The expression intensity of markers related to individual ROIs was used as the input for further analysis.

**Table 1:** Antibody panel.

Antibody	Source	Identifier
E-Cadherin	Fluidigm	3158029D
EpCAM	Fluidigm	3148020D
Vimentin	Fluidigm	3143027D
CD45	Fluidigm	3152018D
CD3	Fluidigm	3710019D
CD4	Fluidigm	3156033D
Foxp3	Fluidigm	3155016D
CD8	Fluidigm	3162034D
CD11c	Fluidigm	3154025D
CD14	Fluidigm	3144025D
CD16	Fluidigm	3146020D
CD163	Abcam	ab87099

### Immunohistochemical Staining

Consecutive, 2.5-μm-thick FFPE sections were manually subjected to immunohistochemical staining. Slides were dewaxed with xylene, rehydrated with graded ethanol and then immunohistochemically stained. The following primary antibodies were used:

Anti-CD163 (1:500, ab87099, Abcam); Anti-CD8 (1:500, ab4055, Abcam); and Anti-FOXP3 (1:300, ab20034, Abcam).

In brief, after tissue sections were dewaxed and rehydrated, antigen retrieval was performed in a pressure cooker for 6 min in Tris-EDTA buffer (pH 9.0). The cells were incubated with 0.3% hydrogen peroxide for 30 min according to the manufacturer’s instructions and then blocked with 3% BSA. The sections were incubated with specific antibodies at 4°C overnight and then labelled with an HRP-conjugated secondary antibody at RT for 1 h. Positive staining was observed with diaminobenzene substrate solution, and then hematoxylin counterstaining was performed.

Under 400x magnification, the absolute number of CD8+ CTLs in the tumor stroma was counted by three of our authors independently, and the average count was used for further analysis. Only the areas with the highest intensity of infiltration within the stroma were selected for evaluation. A similar method was used to determine the absolute number of FoxP3+/CD163+ immune cells; that is, the number of cells was calculated in the areas with the highest degree of infiltration.

### Response to nCRT

In this study, downstaging was defined as a pathological stage lower than the clinical stage based on an imaging examination pretreatment. Patients were categorized into two groups according to the different tumor responses: stage ypT0N0 was defined as the group of patients who achieved pCR (pCR group), and stage ypT1– 4N0/ypTanyN+ was defined as the group of patients who did not achieve pCR (non-pCR group).

### Statistical Analysis

Statistical analysis of the results was performed using (IBM, NY,US), GraphPad Prism 8 (GraphPad Software Inc, CA,US). MCV Viewer(Fluidigm, CA, US), CellProfiler (Whitehead/MIT, MA,US) (16) and HistoCat (UZH, Zurich, Swit) (17) were used to visualize and process the IMC data. Comparisons of the analysed parameters were performed using the nonparametric Mann–Whitney *U* test (for variables on the ordinal scale) or Student’s *t* test (for variables on the interval scale). Spearman rank-order correlation coefficients were used to assess correlations. Logistic regression was used to estimate the odds ratios (ORs) and 95% confidence intervals (CIs) for pCR according to the numbers of CD8+ T cells, M2 macrophages and Tregs after adjusting for age at diagnosis, cT stage (cT3 vs. cT4), cN stage (cN0 vs. cN+) and the chemotherapy regimen (with oxaliplatin vs. without oxaliplatin). Differences and associations were considered statistically significant when P<0.05.

## Results

### Patient Characteristics

A total of 76 patients (6 in the training group, 70 in the validation group) with LARC who were treated with nCRT followed by surgery were enrolled. The characteristics of the two groups of patients are shown in **Table 2**.

**Table 2:** Clinicopathologic parameters.

	Training n = 6	Validation n = 70
Age, median (min,max)	55 (46,61)	57 (28,71)
Gender, n (%)		
male	4 (66.7)	52 (74.3)
female	2 (33.3)	18 (25.7)
T stage, n (%)		
T3	4 (66.7)	47 (67.1)
T4	2 (33.3)	23 (32.9)
N stage, n (%)		
N0	2 (33.3)	16 (35.7)
N+	4 (66.7)	54 (64.3)
differentiation, n (%)		
poor/moderate	4 (66.7)	39 (55.7)
well	2 (33.3)	31 (44.3)
Histological type, n (%)		
ulcerative	4 (66.7)	4 (66.7)
other	2 (33.3)	22 (31.4)
chemotherapy, n (%)		
without oxaliplatin	4 (66.7)	43 (61.5)
with oxaliplatin	2 (33.3)	27 (38.5)

In the training group, to minimize the impact of clinicopathological parameters on the IMC results, a total of 6 patients with matched parameters were enrolled, and the percentage roughly reflected the epidemiological situation. The median age at surgery in the training group was 55 years and ranged from 46 to 61 years. Four patients were male, and two were in an early clinical T stage (cT3) but with lymph node metastasis. Four patients with poorly/moderately differentiated and ulcerative adenocarcinoma were selected, and two received a chemotherapy regimen with oxaliplatin.

In the validation group, the median age at the time of surgery was 57 years (range from 28 to 71 years), and 74.3% of subjects were male. The majority of patients (67.1%) were in clinical T stage 3, and 64.3% were assessed as having nodal involvement on pretreatment CT and MRI scans. Poorly/moderately differentiated (55.7%) and ulcerative (68.6%) adenocarcinomas were the most common pathological types. Twenty-seven (38.5%) patients received chemotherapy containing oxaliplatin. In all, 14 patients (20.0%) who achieved pCR following nCRT were enrolled in this group.

### The Immune Statuses of LARC Patients in the pCR and Non-pCR Groups Who Received nCRT

**Figure 1:**
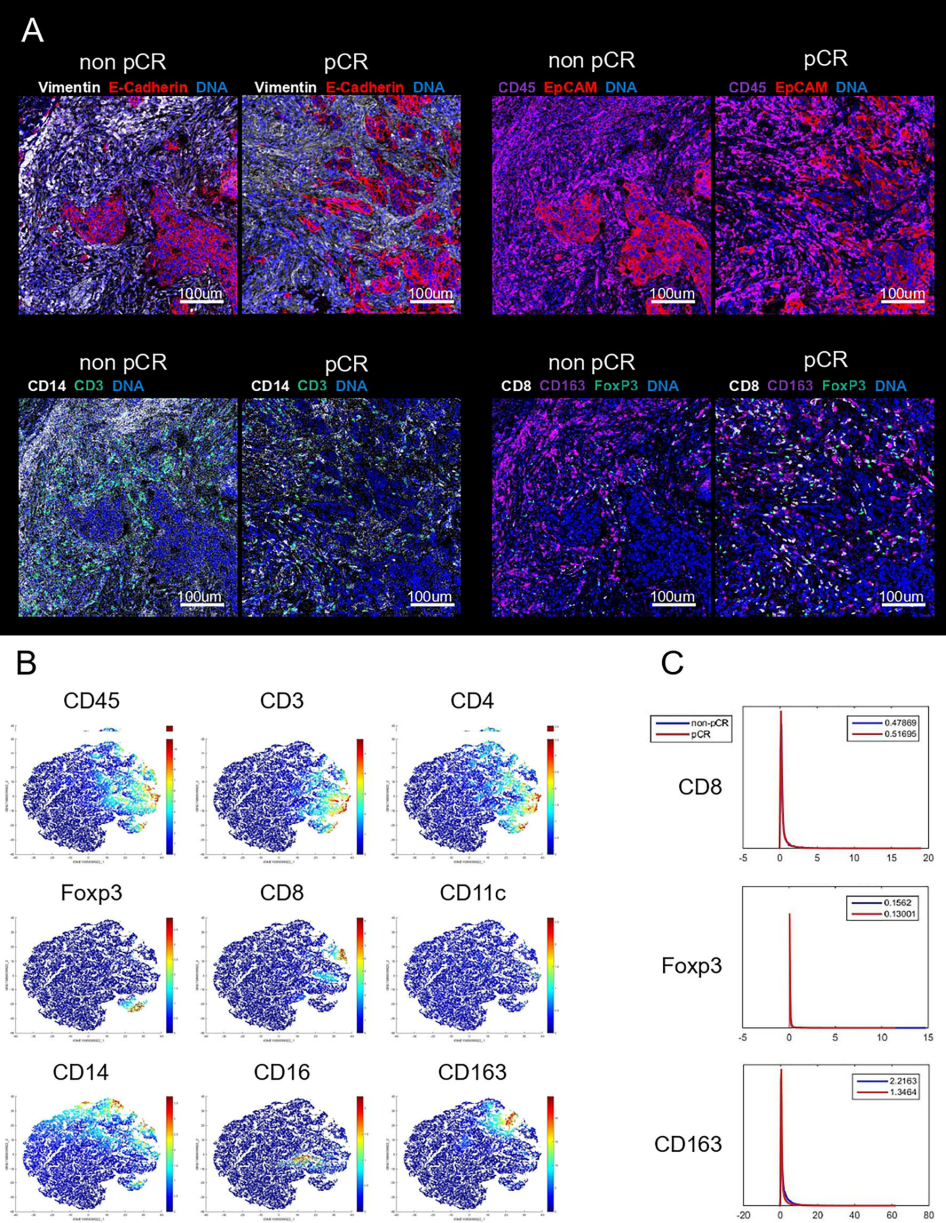
Representative mass cytometry image of LARC tissues in non-pCR and pCR group **(A)**. tSNE map of cells extracted from IMC images illustrating the expression of CD45, CD3, CD4, Foxp3, CD8, CD11c, CD14, CD20 and CD163 **(B)**. The expression intensity of CD8 (upper), Foxp3 (middle), and CD163 (lower) **(C)**.

To comprehensively assess the role of TIICs in predicting the pathological response of LARC patients who receive nCRT, IMC was performed on six tumor tissues from LARC patients (3 who achieved pCR, 3 who did not) before nCRT. Twelve markers were measured per cell per slide. **Figure 1A** shows that TIICs primarily infiltrated the stroma of LARC tissues, and T lymphocytes and macrophages comprised the majority of immune cells in tumors. t-Distributed stochastic neighbor embedding (tSNE) (**Figure 1B**) was also employed to analyze the distributions and characteristics of cells extracted from these images and to identify the coexpression of these markers. tSNE showed similar results, as we observed pseudocolored images directly, and the majority of TIICs consisted of T cells and macrophages. Both CD3+ total T cells and CD4+ T cells(a subset of total T cells) include several subsets, and researches have proven that different roles exerted by different subsets in tumor progression (18). Thus, the anti-tumor CD8+ CTLs and pro-tumor Tregs were selected for further study. As the same reason, CD163+ M2 macrophages, a subset of CD14+ monocytes/macrophages (including anti-cancer M1 macrophages and pro-cancer M2 macrophages) (19), were further validated. The expression intensities of these 3 markers in each ROI were used as input for the statistical analysis. A higher trend of CTLs was observed in patients who achieved pCR, and a higher density of TAMs and Tregs was observed in patients who did not (**Figure 1C**).

### The Associations Between Tumor-Infiltrating CD8+ Cytotoxic T Lymphocytes, CD163+ M2 Macrophages, and Foxp3+ Tregs by Nonparametric Testing

**Figure 2:**
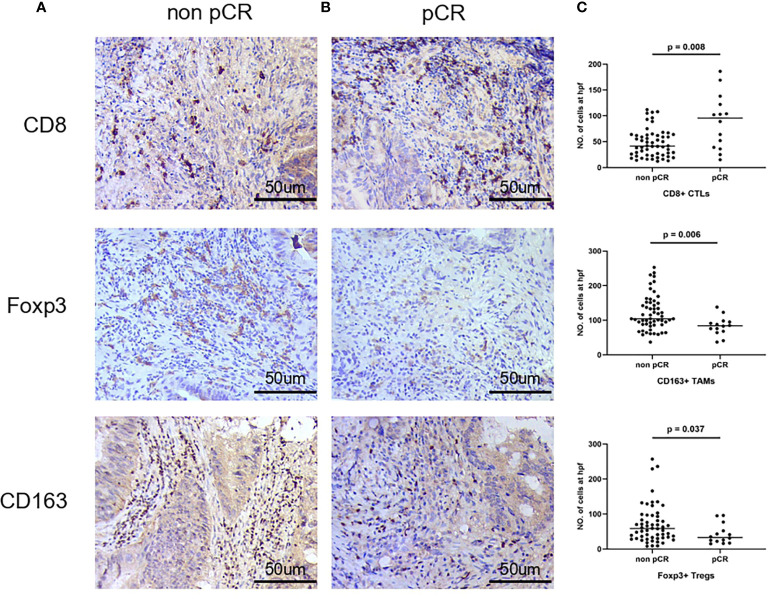
Representative immunohistochemical staining of tumor tissue for CD8, CD163, and Foxp3 in non-pCR group **(A)** and pCR group **(B)** in 400x hpf. **(C)**Frequencies of CD8+, CD163+, and Foxp3+ cells in tumor of two groups.

To further validate the role of CTLs, TAMs and Tregs in predicting the clinicopathological response in LARC patients receiving nCRT, a larger group of LARC tissues was subjected to IHC. The expression levels of CD8, CD163, and Foxp3 in tumor tissues are shown in **Figure 2**. In patients who achieved pCR after nCRT, more intense CTL infiltration was observed, as represented by a lower number of TAMs and Tregs/HPF compared to patients who did not. All these differences were statistically significant (*p* = 0.008, 0.006 and 0.037, respectively).

### The Associations Between Tumor-Infiltrating CD8+ Cytotoxic T Lymphocytes, CD163+ M2 Macrophages, and Foxp3+ Tregs and Clinicopathological Features

**Figure 3:**
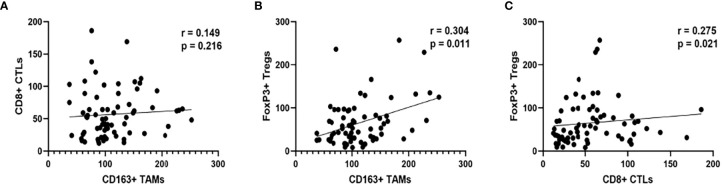
The association between CTLs and TAMs **(A)** Tregs and TAMs **(B)** Tregs and CTLs **(C)**.

As shown in **Figure 3**, Foxp3+ Tregs in tumor tissues were positively correlated with CD163+ macrophages (*p* = 0.011) and CD8+ T cells (*p* = 0.021). Nevertheless, no significant correlation between CD163+ macrophages and CD8+ T lymphocytes was observed.

We further analysed the association between tumor-infiltrating CTLs, TAMs and Tregs and the clinicopathological features of LARC patients. Our data showed a strong association between the number of TAMs and cN stage (*p* = 0.026), with higher numbers of CD163-positive TAMs in tumors with lymph node metastasis. However, no association between clinical T stage and the number of TAMs was found (**Table 3**).

**Table 3:** The association between tumor-infiltrating CD8+ CTLs, CD163+ M2 TAMs, Foxp3+ Tregs and clinicopathological features.

	CD8+ CTL		CD163+ M2 macrophage		Foxp3+ Treg	
	median (IQR)	*P*	median (IQR)	*P*	median (IQR)	*P*
Age		0.700		0.108		0.854
<55 N=30	46 (34, 65)		92 (71, 117)		50 (29,83)	
≥55 N=40	49 (23, 73)		113 (84, 149)		52 (29, 92)	
Gender		0.667		0.914		0.872
male N=52	45 (27, 67)		100 (85, 134)		52 (30, 82)	
female N=18	52 (19, 73)		104 (64, 157)		47 (26, 104)	
differentiation, n (%)		0.321		0.343		0.955
poor/moderate N=48	45 (25, 64)		99 (78, 134)		53 (28, 95)	
well N=22	57 (26, 104)		118 (81, 156)		46 (31, 78)	
Histological type, n (%)		0.295		0.896		0.266
ulcerative	55 (35, 68)		99 (72, 143)		53 (30, 96)	
N=39 other N=31	40 (23, 64)		102 (87, 138)		48 (25, 70)	
T stage		0.431		0.750		0.644
T3 N=47	42 (27, 65)		101 (85, 143)		53 (33, 81)	
T4 N=23	53 (25, 89)		99 (76, 138)		43 (25, 103)	
N stage		0.260		0.026		0.418
N0 N=24	39 (17, 73)		89 (67, 116)		42 (25, 77)	
N+ N=46	52 (29, 64)		108 (85, 145)		53 (30, 95)	

### The Associations Between Tumor-Infiltrating CD8+ Cytotoxic T Lymphocytes, CD163+ M2 Macrophages, Foxp3+ Tregs, and the Response to nCRT by Logistic Regression

**Table 4** shows the results of the univariate and multivariate logistic regression analyses of clinicopathological and immunological features with respect to the patients’ responses to nCRT. The univariate analysis revealed that a high baseline number of tumor-infiltrating CD8+ CTLs was significantly associated with pCR (OR = 1.030; 95% CI: 1.011~1.048, *p* = 0.002), whereas high expression of CD163+ TAMs was significantly associated with no pCR (OR = 0.975; 95% CI: 0.955~0.995, *p* = 0.015). After adjusting for demographic factors, including age at diagnosis, sex, pathological type, clinical stage, and chemotherapy regimen, in the multivariate logistic regression analysis, the results showed that a high level of CD8+ CTLs was an independent factor associated with pCR (OR=1.042; 95% CI: 1.015~1.070, *p* = 0.002). In contrast, a high level of TAMs emerged as an independent factor associated with no pCR (OR=0.969; 95% CI: 0.941~0.998, p=0.036). However, Tregs showed no statistical significance in either the univariate (OR=0.982; 95% CI: 0.962~1.002, *p* = 0.070) or multivariate (OR=0.978; 95% CI: 0.949~1.007, p=0.139) logistic regression analysis although the density of Tregs was negatively associated with pCR in the nonparametric test (*p* = 0.037).

**Table 4:** Logistic regression analysis of immunological and clinicopathological features with respect to LACR patients’ clinical response to nCRT.

	Univariate analysis			Multivariate analysis		
	*OR*	95%Cl	*p-value*	*OR*	95%Cl	*p-value*
CD8	1.030	1.011~1.048	0.002	1.042	1.015~1.070	0.002
CD163	0.975	0.955~0.995	0.015	0.969	0.941~0.998	0.036
FoxP3	0.982	0.962~1.002	0.070	0.978	0.949~1.007	0.139
Age <55 ≥55	1.000 1.435	0.443~4.647	0.547	1.000 0.485	0.065~3.608	0.480
Gender male female	1.000 0.745	0.183~3.044	0.682	1.000 0.677	0.099~4.632	0.691
differentiation poor/moderate well	1.000 1.875	0.561~6.268	0.307	1.000 1.174	0.141~9.770	0.882
Histological type ulcerative other	1.000 1.075	0.330~3.508	0.904	1.000 2.478	0.315~19.484	0.389
cT stage cT3 cT4	1.000 0.711	0.201~2.515	0.597	1.000 0.728	0.097~5.486	0.758
cN stage cN0 cN+	1.000 2.294	0.696~7.560	0.172	1.000 4.476	0.531~37.763	0.168
chemotherapy with oxaliplatin without oxaliplatin	1.000 2.759	0.681~11.175	0.155	1.000 6.236	0.622~62.529	0.120

## Discussion

In this study, we developed a 12-antibody panel containing the markers of the major TIIC subset to determine whether the immune status differs between LARC patients who respond to nCRT differently. A higher expression intensity of CD8 cells was observed in patients who achieved pCR, and a higher density of TAMs and Tregs was observed in those who did not. To further investigate the relationship between the densities of CD8+ CTLs, CD163+ TAMs and Foxp3+ Tegs in the local TME before nCRT and treatment response in LARC patients, a larger group of LARC patients were enrolled as an independent validation group. In the multiple logistic regression analysis, CTLs and TAMs were revealed as independent predictors for a good response to nCRT, suggesting that CTLs and TAMs play key immunoregulatory roles in RCT-associated antitumour processes. Therefore, CTL and TAM infiltration levels are expected to become potential predictive biomarkers of nCRT sensitivity in LARC patients.

Since recommended as the standard treatment for LARC, nCRT was proven to significantly improve pCR rates (1, 20). pCR after nCRT was proposed as a key prognostic indicator for better outcomes in LARC (3, 21). In a meta-analysis which included 1913 patients by Zorcolos et al, Patients with pCR had a much lower rate of local tumor recurrence and distant metastasis. Besides, OS was longer for those achieved pCR (92.9% for pCR versus 73.4% for non-pCR at 5^th^ year) (3). The similar results were seen in a German Rectal Trial (CAO/ARO/AIO-94 trial), 86% of patients with a pCR remained free of disease at the end of 5 years, whereas only 63% of patients without pCR (22). However, as in present study, only minority of LARC patients achieved pCR after received nCRT, up to 80% of the patients experienced incomplete tumor regression or even progression after nCRT (4). The current study was dedicated to explore the relationship of preoperative level of TIICs and efficiency of nCRT, and aid the therapeutic strategy-making.

In this study, we demonstrated that a higher CD8 + TIL count before nCRT was associated with a better pathologic response, which is reasonably explained by experimental results showing that CTLs play a crucial role in the antitumour effects of cytotoxic drugs and radiation (23, 24). Recently, it has been reported that several molecules, including calreticulin, HMGB1, and ATP, released by tumor cells are responsible for chemotherapy-induced anticancer immune responses (10, 25). These molecules mediate the proliferation and activation of CTLs *via* multiple pathways (26). Radiation therapy has also shown its antitumour effect by inducing neoantigens from tumor cells (10), similar to the role of chemotherapy, and this effect may be significantly weakened after the depletion of CTLs.

M1 macrophages and M2 macrophages play opposing roles in tumor development (19, 27). Recent studies have focused on CD163 staining in representative LARC tissues to investigate the clinical significance of TAMs (28, 29). Studies have shown that a higher density of M2 macrophages strongly predicts a poor response to nCRT and shorter OS and DFS in colorectal cancer (CRC) patients (28, 29). In our study, the number of TAMs/HPF was an independent predictor for the response to nCRT, as intense TAM staining was associated with a poor pathological response. Mechanistically, numerous cytokines and pathways, such as the IL-6R/STAT3/miR-204-5p pathway, were found to be involved in chemoresistance mediated by macrophages (30, 31). After radiotherapy, altered inflammatory cytokines (such as IL-6, IL-10, and TNF) lead to the recruitment of TAMs with a tissue reparative phenotype and contribute to tumor recurrence and metastasis (31). However, the studies above focused on the dynamics and effect of TAMs after radiation, and the predictive role of TAMs is poorly understood. Our present study showed that TAMs before nCRT play an important role in CRT resistance. Our results may partially be explained by the positive crosstalk between TAMs and cells related to immune suppression, including Th2 cells, cancer‐associated fibroblasts, Tregs, and MDSCs, and the negative crosstalk with CTLs (19, 32). This hypothesis needs to be further investigated in future studies.

A low number of Tregs was demonstrated to be strongly associated with pCR and better clinical outcomes in several cancers (9, 33, 34); therefore, it is shocking that no association between Tregs and clinical response was observed in the logistic regression analysis. The selection of truly representative target areas is a challenge common to all studies using biopsy samples and can be particularly difficult when using tissue from pretreated patients with significant variation in treatment response. For all studies using biopsy samples, whether the target is representative is a common problem (35). Although biopsies are carefully sampled by experienced endoscopists, it cannot be ruled out that the lack of a correlation between Treg density in tumors may be a problem associated with sampling. However, the number of Tregs was significantly different between the two groups *via* the Mann–Whitney *U* test, which proved the robustness of our study.

In this study, we observed that a high infiltration rate of Tregs was associated with a high infiltration rate of CTLs. Our findings could be explained by the fact that similar mechanisms are shared by CTLs and Tregs when infiltrating tumors (36, 37). For T cells to initiate extravasation, they must interact the CD44, CD62L or PSGL with endothelial selectin. All these molecules are expressed on the membranes of Tregs and activated CTLs. In addition, most of the chemokine receptors that mediate CD8+ T cell extravasation, such as CCR5, CXCR3 and CXCR6, are expressed on Tregs. As such, it is logical that Tregs and CTLs co-infiltrate a tumor. A positive correlation between TAMs and Tregs was also found. As discussed above, TAMs can directly recruit Tregs and activate them to establish the immunosuppressive TME (19). Numerous chemokines, such as CCL20 and CCL22, have been reported to participate in this process (38).

Regarding clinicopathological features, a higher density of TAMs was correlated with a positive lymph node stage in our study. As the major component of TIICs, TAMs were well-known to promote distant metastasis by multiple mechanisms (39, 40), and current studies found that the tumor cells present in lymph nodes maybe an important source of systemic metastasis (41, 42). In the most recent study by Chen et al, TAMs were found to significantly higher in breast primary tumor with lymph node metastasis compared with tumor tissues without lymph node involvement (43).

As the discussion above, the TAMs was found to be associated with nodal stage in present study, but no statistical relationship was found between the TAMs and tumor size, these result aroused our interest in exploring the mechanism underlying the role of M2 TAMs in lymph node metastasis in the early T stage.

This study had some limitations: first, this was a relatively small cohort of patients; thus, some clinically relevant factors, such as cT classification, failed to show significant differences. Therefore, these results must be confirmed in larger samples and through multicenter studies. Second, the clinicopathological parameters included are not comprehensive enough, which may lead to inefficiency of the model. In addition, the analyses performed were only correlation analyses, and further experimental studies are needed to explore the potential mechanism of these correlations.

Based on our study, these positive immunoregulatory features in preoperative evaluation may be suggestive for the need of combining current neoadjuvant regimens with an immune-modulating therapy to achieve better therapeutic outcome and survival in cancer treatment. However, as a correlation analyses with a relatively small cohort of patients, it still a long way to transform the results of current study into clinical applications.

In conclusion, this study proved that Tregs in tumor tissues were positively correlated with TAMs and CTLs. The density of tumor-infiltrating TAMs is a significant factor for lymph node metastasis. These findings suggest that TAMs and CTLs are predictive markers for the sensitivity of nCRT and might aid in clinical decision-making regarding the delivery of improved therapies for LARC. We believe that our data will provide the foundation for developing new prognostic biomarkers and improving the nCRT treatment outcomes of LARC patients. Besides, as total neoadjuvant therapy(TNT) with higher rate of achieving pCR emerging as an alternative treatment to LARC, the predictive role of CTLs, TAMs and Trgs play in LARC patients received TNT aroused our great interest and will be the next focus.

## Data Availability Statement

The original contributions presented in the study are included in the article. Further inquiries can be directed to the corresponding authors.

## Ethics Statement

The studies involving human participants were reviewed and approved by Ethics Committee of the Fifth affiliated Hospital of Sun Yat-sen University and Harbin Medical University Cancer Hospital. The patients/participants provided their written informed consent to participate in this study.

## Author Contributions

HZ, BZ and QZ conceived and designed the experiments. YY, WT, LQS, PL, XG, and LS conducted and analyzed the data. YY and WT wrote this manuscript. All authors contributed to the article and approved the submitted version.

## Funding

This study was financially supported from Guangdong Basic and Applied Basic Research Foundation (Grant Number: 2021A1515010421), National Natural Science Foundation of China (Grant Number. 31571417) and Natural Science Foundation of Zhejiang Province (Grant Number. LZ21H160006).

## Conflict of Interest

The authors declare that the research was conducted in the absence of any commercial or financial relationships that could be construed as a potential conflict of interest.

## Publisher’s Note

All claims expressed in this article are solely those of the authors and do not necessarily represent those of their affiliated organizations, or those of the publisher, the editors and the reviewers. Any product that may be evaluated in this article, or claim that may be made by its manufacturer, is not guaranteed or endorsed by the publisher.
